# 6,12-Dihydro­dipyrido[1,2-*a*:1′,2′-*d*]pyrazinium bis­(perchlorate)

**DOI:** 10.1107/S1600536809032528

**Published:** 2009-08-22

**Authors:** Nam-Ho Kim, Kwang Ha

**Affiliations:** a School of Applied Chemical Engineering, the Research Institute of Catalysis, Chonnam National University, Gwangju 500-757, Republic of Korea

## Abstract

In the title compound, C_12_H_12_N_2_^2+^·2ClO_4_^−^, the dihedral angle between the two outer pyridine rings of the dication is 44.8 (1)°. In the crystal, weak intermolecular C—H⋯O hydrogen bonds occur.

## Related literature

For the crystal structure of (C_12_H_12_N_2_)Br_2_, see: Bryce *et al.* (1985 ▶). For a MNDO (modified neglect of diatomic overlap) study of dipyridopyrazinium and related cations, see: Eaves *et al.* (1986 ▶).
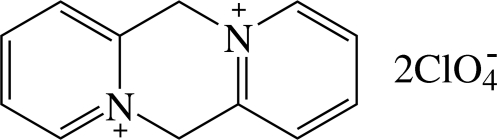


## Experimental

### 

#### Crystal data

C_12_H_12_N_2_^2+^·2ClO_4_^−^
*M* *_r_* = 383.14
Monoclinic, 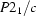

*a* = 8.1632 (8) Å
*b* = 13.9396 (14) Å
*c* = 13.5903 (13) Å
β = 96.023 (2)°
*V* = 1537.9 (3) Å^3^
*Z* = 4
Mo *K*α radiation
μ = 0.47 mm^−1^
*T* = 296 K
0.22 × 0.16 × 0.10 mm

#### Data collection

Bruker SMART 1000 CCD diffractometer
Absorption correction: multi-scan (*SADABS*; Bruker, 2000 ▶) *T*_min_ = 0.646, *T*_max_ = 0.954
11269 measured reflections
3809 independent reflections
1871 reflections with *I* > 2σ(*I*)
*R*_int_ = 0.068

#### Refinement

*R*[*F* ^2^ > 2σ(*F* ^2^)] = 0.058
*wR*(*F* ^2^) = 0.163
*S* = 1.06
3809 reflections
217 parameters
H-atom parameters constrained
Δρ_max_ = 0.39 e Å^−3^
Δρ_min_ = −0.48 e Å^−3^

### 

Data collection: *SMART* (Bruker, 2000 ▶); cell refinement: *SAINT* (Bruker, 2000 ▶); data reduction: *SAINT*; program(s) used to solve structure: *SHELXS97* (Sheldrick, 2008 ▶); program(s) used to refine structure: *SHELXL97* (Sheldrick, 2008 ▶); molecular graphics: *ORTEP-3* (Farrugia, 1997 ▶) and *PLATON* (Spek, 2009 ▶); software used to prepare material for publication: *SHELXL97*.

## Supplementary Material

Crystal structure: contains datablocks global, I. DOI: 10.1107/S1600536809032528/om2266sup1.cif

Structure factors: contains datablocks I. DOI: 10.1107/S1600536809032528/om2266Isup2.hkl

Additional supplementary materials:  crystallographic information; 3D view; checkCIF report

## Figures and Tables

**Table 1:** Hydrogen-bond geometry (Å, °)

*D*—H⋯*A*	*D*—H	H⋯*A*	*D*⋯*A*	*D*—H⋯*A*
C1—H1⋯O1	0.93	2.49	3.358 (6)	156
C2—H2⋯O1^i^	0.93	2.57	3.160 (5)	122
C4—H4⋯O6^i^	0.93	2.54	3.228 (5)	132
C6—H6*B*⋯O3^ii^	0.97	2.56	3.343 (5)	137
C7—H7⋯O6^iii^	0.93	2.45	3.249 (5)	144
C9—H9⋯O7^iv^	0.93	2.44	3.190 (5)	138

Symmetry codes: (i) 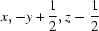; (ii) 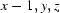; (iii) 
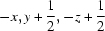
; (iv) 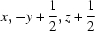.

**Fig. 1.**
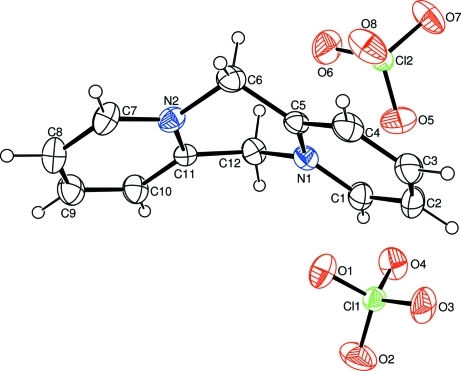
The structure of the title compound, with displacement ellipsoids drawn at the 30% probability level for non-H atoms.

**Fig. 2.**
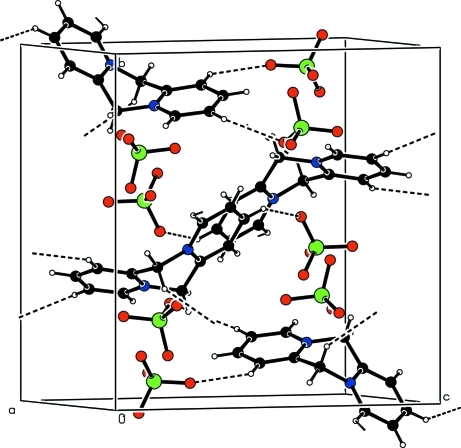
View of the unit-cell contents of the title compound. Hydrogen-bond interactions are drawn with dashed lines.

## supplementary crystallographic information

### Comment

The asymmetric unit of the title compound, C_12_H_12_N_2_^2+^.2ClO_4_^-^, consists of a 6,12-dihydrodipyrido[1,2-*a*:1',2'-*d*]pyrazinium dication and two perchlorate counter-anions (Fig. 1). In the dication, two pyridine rings are linked by two methylene groups and the bridgehead N atoms are disposed on the opposite side of the central six-membered ring which adopts an eclipsed boat conforamtion. The two methylene C atoms (C6 and C12) lie practically on the pyridine ring planes with the largest deviations 0.046 (6) Å (C6) and 0.023 (6) Å (C12) from the respective least-squares planes, and the dihedral angles between these planes is 44.8 (1)°. The geometry of the ClO_4_^-^ anions is nearly tetrahedral with the O—Cl—O bond angles of 107.4 (2)°–112.5 (2)°, and the Cl—O bond distances are almost equal (1.416 (3)–1.426 (3) Å). The compound displays intermolecular C—H···O hydrogen bonds (Table 1 and Fig. 2). There may also be weak intermolecular π-π interactions between adjacent pyridine rings, with a shortest centroid-centroid distance of 5.057 (2) Å.

### Experimental

Single crystals of the title compound were unexpectedly obtained as a byproduct of an attempted preparation of an Mn(II) complex by reacting 2-(chloromethyl)pyridine hydrochloride (0.99 g, 6.04 mmol), 1,6-diaminohexane (0.17 g, 1.46 mmol), NaOH (for adjustment of pH 7–8) and Mn(ClO_4_)_2_.6H_2_O (0.37 g, 1.02 mmol) in EtOH (10 ml) and H_2_O (5 ml) for 2 h at 60 °C. Crystals suitable for X-ray analysis were obtained by slow evaporation from a CH_3_CN solution of the orange reaction product.

### Refinement

H atoms were positioned geometrically and allowed to ride on their respective carrier atoms [C—H = 0.93 (*sp*^2^) or 0.97 Å (*sp*^3^) and *U*_iso_(H) = 1.2*U*_eq_(C)].

### Crystal data

**Table:**

C_12_H_12_N_2_^2+^·2ClO_4_^−^	*F*(000) = 784
*M**_r_* = 383.14	*D*_x_ = 1.655 Mg m^−^^3^
Monoclinic, *P*2_1_/*c*	Mo *K*α radiation, λ = 0.71073 Å
Hall symbol: -P 2ybc	Cell parameters from 2227 reflections
*a* = 8.1632 (8) Å	θ = 2.5–23.7°
*b* = 13.9396 (14) Å	µ = 0.47 mm^−^^1^
*c* = 13.5903 (13) Å	*T* = 296 K
β = 96.023 (2)°	Block, colorless
*V* = 1537.9 (3) Å^3^	0.22 × 0.16 × 0.10 mm
*Z* = 4	

### Data collection

**Table:**

Bruker SMART 1000 CCD diffractometer	3809 independent reflections
Radiation source: fine-focus sealed tube	1871 reflections with *I* > 2σ(*I*)
graphite	*R*_int_ = 0.068
φ and ω scans	θ_max_ = 28.3°, θ_min_ = 2.1°
Absorption correction: multi-scan (*SADABS*; Bruker, 2000)	*h* = −10→10
*T*_min_ = 0.646, *T*_max_ = 0.954	*k* = −18→16
11269 measured reflections	*l* = −15→18

### Refinement

**Table:**

Refinement on *F*^2^	Primary atom site location: structure-invariant direct methods
Least-squares matrix: full	Secondary atom site location: difference Fourier map
*R*[*F*^2^ > 2σ(*F*^2^)] = 0.058	Hydrogen site location: inferred from neighbouring sites
*wR*(*F*^2^) = 0.163	H-atom parameters constrained
*S* = 1.06	*w* = 1/[σ^2^(*F*_o_^2^) + (0.0561*P*)^2^ + 0.3329*P*] where *P* = (*F*_o_^2^ + 2*F*_c_^2^)/3
3809 reflections	(Δ/σ)_max_ < 0.001
217 parameters	Δρ_max_ = 0.39 e Å^−^^3^
0 restraints	Δρ_min_ = −0.48 e Å^−^^3^

### Special details

**Table:**

Geometry. All e.s.d.'s (except the e.s.d. in the dihedral angle between two l.s. planes) are estimated using the full covariance matrix. The cell e.s.d.'s are taken into account individually in the estimation of e.s.d.'s in distances, angles and torsion angles; correlations between e.s.d.'s in cell parameters are only used when they are defined by crystal symmetry. An approximate (isotropic) treatment of cell e.s.d.'s is used for estimating e.s.d.'s involving l.s. planes.
Refinement. Refinement of *F*^2^ against ALL reflections. The weighted *R*-factor *wR* and goodness of fit *S* are based on *F*^2^, conventional *R*-factors *R* are based on *F*, with *F* set to zero for negative *F*^2^. The threshold expression of *F*^2^ > σ(*F*^2^) is used only for calculating *R*-factors(gt) *etc*. and is not relevant to the choice of reflections for refinement. *R*-factors based on *F*^2^ are statistically about twice as large as those based on *F*, and *R*- factors based on ALL data will be even larger.

### Fractional atomic coordinates and isotropic or equivalent isotropic displacement parameters (Å^2^)

**Table:**

	*x*	*y*	*z*	*U*_iso_*/*U*_eq_	
N1	0.4245 (4)	0.3340 (2)	0.2124 (2)	0.0413 (8)	
N2	0.1643 (4)	0.4419 (2)	0.2709 (2)	0.0424 (8)	
C1	0.5777 (5)	0.3093 (3)	0.1934 (3)	0.0512 (11)	
H1	0.6517	0.2840	0.2435	0.061*	
C2	0.6240 (6)	0.3214 (3)	0.1007 (3)	0.0592 (12)	
H2	0.7284	0.3025	0.0867	0.071*	
C3	0.5151 (6)	0.3618 (3)	0.0275 (3)	0.0584 (12)	
H3	0.5465	0.3718	−0.0356	0.070*	
C4	0.3592 (5)	0.3872 (3)	0.0490 (3)	0.0518 (11)	
H4	0.2849	0.4144	0.0004	0.062*	
C5	0.3140 (4)	0.3723 (2)	0.1422 (3)	0.0360 (8)	
C6	0.1484 (5)	0.3943 (3)	0.1727 (3)	0.0486 (10)	
H6A	0.0892	0.4359	0.1240	0.058*	
H6B	0.0860	0.3353	0.1759	0.058*	
C7	0.0714 (5)	0.5186 (3)	0.2886 (3)	0.0528 (11)	
H7	−0.0036	0.5428	0.2386	0.063*	
C8	0.0869 (6)	0.5607 (3)	0.3796 (3)	0.0607 (12)	
H8	0.0235	0.6140	0.3919	0.073*	
C9	0.1978 (5)	0.5233 (3)	0.4535 (3)	0.0582 (12)	
H9	0.2083	0.5505	0.5163	0.070*	
C10	0.2924 (5)	0.4459 (3)	0.4336 (3)	0.0497 (10)	
H10	0.3685	0.4211	0.4828	0.060*	
C11	0.2753 (4)	0.4047 (2)	0.3413 (2)	0.0334 (8)	
C12	0.3709 (5)	0.3198 (3)	0.3127 (3)	0.0444 (10)	
H12A	0.4667	0.3107	0.3604	0.053*	
H12B	0.3031	0.2627	0.3130	0.053*	
Cl1	0.93531 (13)	0.21962 (7)	0.40847 (7)	0.0483 (3)	
O1	0.7805 (4)	0.2646 (2)	0.4167 (2)	0.0735 (9)	
O2	1.0582 (4)	0.2624 (3)	0.4763 (3)	0.1006 (13)	
O3	0.9764 (4)	0.2311 (2)	0.3100 (2)	0.0798 (10)	
O4	0.9247 (4)	0.1204 (2)	0.4313 (2)	0.0759 (10)	
Cl2	0.38056 (13)	0.06673 (7)	0.22970 (8)	0.0508 (3)	
O5	0.5488 (4)	0.0854 (3)	0.2602 (3)	0.0955 (12)	
O6	0.2971 (4)	0.0624 (3)	0.3163 (2)	0.0988 (13)	
O7	0.3618 (4)	−0.0210 (2)	0.1771 (2)	0.0821 (11)	
O8	0.3058 (5)	0.1403 (2)	0.1684 (2)	0.0844 (11)	

### Atomic displacement parameters (Å^2^)

**Table:**

	*U*^11^	*U*^22^	*U*^33^	*U*^12^	*U*^13^	*U*^23^
N1	0.047 (2)	0.0404 (18)	0.0363 (17)	−0.0034 (15)	0.0031 (15)	−0.0021 (14)
N2	0.0370 (18)	0.053 (2)	0.0378 (17)	−0.0020 (15)	0.0059 (15)	0.0048 (15)
C1	0.048 (3)	0.052 (3)	0.054 (3)	0.0070 (19)	0.006 (2)	−0.007 (2)
C2	0.054 (3)	0.078 (3)	0.048 (3)	0.002 (2)	0.020 (2)	−0.012 (2)
C3	0.065 (3)	0.068 (3)	0.044 (2)	−0.011 (2)	0.014 (2)	−0.011 (2)
C4	0.063 (3)	0.060 (3)	0.031 (2)	−0.009 (2)	0.000 (2)	0.0016 (19)
C5	0.038 (2)	0.040 (2)	0.0285 (18)	−0.0069 (17)	0.0014 (16)	−0.0012 (15)
C6	0.048 (3)	0.061 (3)	0.036 (2)	−0.006 (2)	−0.0005 (19)	−0.0014 (19)
C7	0.044 (3)	0.062 (3)	0.053 (3)	0.015 (2)	0.010 (2)	0.009 (2)
C8	0.065 (3)	0.059 (3)	0.061 (3)	0.018 (2)	0.021 (3)	−0.003 (2)
C9	0.067 (3)	0.065 (3)	0.044 (2)	−0.001 (2)	0.015 (2)	−0.007 (2)
C10	0.057 (3)	0.057 (3)	0.034 (2)	0.004 (2)	0.0051 (19)	−0.0004 (19)
C11	0.034 (2)	0.0347 (19)	0.0311 (18)	−0.0020 (15)	0.0029 (16)	0.0035 (15)
C12	0.054 (3)	0.044 (2)	0.035 (2)	0.0008 (19)	0.0062 (19)	0.0034 (17)
Cl1	0.0477 (6)	0.0519 (6)	0.0458 (6)	0.0002 (5)	0.0080 (5)	−0.0004 (5)
O1	0.062 (2)	0.088 (2)	0.073 (2)	0.0230 (18)	0.0171 (17)	0.0126 (18)
O2	0.086 (3)	0.098 (3)	0.108 (3)	−0.009 (2)	−0.035 (2)	−0.029 (2)
O3	0.103 (3)	0.083 (2)	0.061 (2)	−0.001 (2)	0.046 (2)	0.0076 (18)
O4	0.106 (3)	0.0527 (19)	0.071 (2)	0.0025 (18)	0.020 (2)	0.0110 (16)
Cl2	0.0563 (7)	0.0471 (6)	0.0472 (6)	0.0066 (5)	−0.0020 (5)	−0.0077 (5)
O5	0.055 (2)	0.120 (3)	0.107 (3)	−0.005 (2)	−0.015 (2)	−0.016 (2)
O6	0.085 (3)	0.166 (4)	0.048 (2)	0.000 (2)	0.0160 (19)	−0.012 (2)
O7	0.117 (3)	0.0503 (19)	0.072 (2)	0.0157 (18)	−0.021 (2)	−0.0168 (16)
O8	0.120 (3)	0.0476 (18)	0.078 (2)	0.0072 (18)	−0.026 (2)	0.0011 (16)

### Geometric parameters (Å, °)

**Table:**

N1—C1	1.348 (5)	C7—H7	0.9300
N1—C5	1.352 (4)	C8—C9	1.382 (6)
N1—C12	1.488 (4)	C8—H8	0.9300
N2—C7	1.346 (5)	C9—C10	1.370 (5)
N2—C11	1.351 (4)	C9—H9	0.9300
N2—C6	1.484 (5)	C10—C11	1.372 (5)
C1—C2	1.364 (6)	C10—H10	0.9300
C1—H1	0.9300	C11—C12	1.492 (5)
C2—C3	1.383 (6)	C12—H12A	0.9700
C2—H2	0.9300	C12—H12B	0.9700
C3—C4	1.382 (6)	Cl1—O2	1.420 (3)
C3—H3	0.9300	Cl1—O4	1.421 (3)
C4—C5	1.371 (5)	Cl1—O3	1.423 (3)
C4—H4	0.9300	Cl1—O1	1.426 (3)
C5—C6	1.487 (5)	Cl2—O5	1.416 (3)
C6—H6A	0.9700	Cl2—O7	1.417 (3)
C6—H6B	0.9700	Cl2—O8	1.418 (3)
C7—C8	1.363 (6)	Cl2—O6	1.421 (3)
			
C1—N1—C5	122.0 (3)	C7—C8—C9	119.2 (4)
C1—N1—C12	120.6 (3)	C7—C8—H8	120.4
C5—N1—C12	117.4 (3)	C9—C8—H8	120.4
C7—N2—C11	121.7 (3)	C10—C9—C8	119.6 (4)
C7—N2—C6	121.3 (3)	C10—C9—H9	120.2
C11—N2—C6	117.0 (3)	C8—C9—H9	120.2
N1—C1—C2	119.8 (4)	C9—C10—C11	120.3 (4)
N1—C1—H1	120.1	C9—C10—H10	119.9
C2—C1—H1	120.1	C11—C10—H10	119.9
C1—C2—C3	119.7 (4)	N2—C11—C10	118.9 (3)
C1—C2—H2	120.1	N2—C11—C12	116.7 (3)
C3—C2—H2	120.1	C10—C11—C12	124.4 (3)
C4—C3—C2	119.3 (4)	N1—C12—C11	110.2 (3)
C4—C3—H3	120.4	N1—C12—H12A	109.6
C2—C3—H3	120.4	C11—C12—H12A	109.6
C5—C4—C3	120.0 (4)	N1—C12—H12B	109.6
C5—C4—H4	120.0	C11—C12—H12B	109.6
C3—C4—H4	120.0	H12A—C12—H12B	108.1
N1—C5—C4	119.2 (3)	O2—Cl1—O4	108.8 (2)
N1—C5—C6	116.3 (3)	O2—Cl1—O3	110.1 (2)
C4—C5—C6	124.5 (4)	O4—Cl1—O3	109.87 (19)
N2—C6—C5	110.3 (3)	O2—Cl1—O1	109.6 (2)
N2—C6—H6A	109.6	O4—Cl1—O1	109.6 (2)
C5—C6—H6A	109.6	O3—Cl1—O1	108.8 (2)
N2—C6—H6B	109.6	O5—Cl2—O7	110.9 (2)
C5—C6—H6B	109.6	O5—Cl2—O8	112.5 (2)
H6A—C6—H6B	108.1	O7—Cl2—O8	108.15 (19)
N2—C7—C8	120.3 (4)	O5—Cl2—O6	107.4 (2)
N2—C7—H7	119.8	O7—Cl2—O6	110.2 (2)
C8—C7—H7	119.8	O8—Cl2—O6	107.6 (2)
			
C5—N1—C1—C2	−1.1 (6)	C11—N2—C7—C8	−0.4 (6)
C12—N1—C1—C2	179.0 (3)	C6—N2—C7—C8	179.4 (4)
N1—C1—C2—C3	2.1 (6)	N2—C7—C8—C9	−0.5 (6)
C1—C2—C3—C4	−1.6 (6)	C7—C8—C9—C10	1.2 (6)
C2—C3—C4—C5	0.1 (6)	C8—C9—C10—C11	−1.1 (6)
C1—N1—C5—C4	−0.5 (5)	C7—N2—C11—C10	0.5 (5)
C12—N1—C5—C4	179.5 (3)	C6—N2—C11—C10	−179.2 (3)
C1—N1—C5—C6	178.8 (3)	C7—N2—C11—C12	−179.9 (3)
C12—N1—C5—C6	−1.3 (5)	C6—N2—C11—C12	0.3 (4)
C3—C4—C5—N1	0.9 (6)	C9—C10—C11—N2	0.2 (6)
C3—C4—C5—C6	−178.2 (4)	C9—C10—C11—C12	−179.3 (4)
C7—N2—C6—C5	136.2 (4)	C1—N1—C12—C11	137.8 (3)
C11—N2—C6—C5	−44.1 (4)	C5—N1—C12—C11	−42.2 (4)
N1—C5—C6—N2	44.5 (4)	N2—C11—C12—N1	42.6 (4)
C4—C5—C6—N2	−136.4 (4)	C10—C11—C12—N1	−137.9 (4)

### Hydrogen-bond geometry (Å, °)

**Table:**

*D*—H···*A*	*D*—H	H···*A*	*D*···*A*	*D*—H···*A*
C1—H1···O1	0.93	2.49	3.358 (6)	156
C2—H2···O1^i^	0.93	2.57	3.160 (5)	122
C4—H4···O6^i^	0.93	2.54	3.228 (5)	132
C6—H6B···O3^ii^	0.97	2.56	3.343 (5)	137
C7—H7···O6^iii^	0.93	2.45	3.249 (5)	144
C9—H9···O7^iv^	0.93	2.44	3.190 (5)	138

Symmetry codes: (i) *x*, −*y*+1/2, *z*−1/2; (ii) *x*−1, *y*, *z*; (iii) −*x*, *y*+1/2, −*z*+1/2; (iv) *x*, −*y*+1/2, *z*+1/2.
